# The Effect of the Missouri WISEWOMAN Program on Control of Hypertension, Hypercholesterolemia, and Elevated Blood Glucose Among Low-Income Women

**DOI:** 10.5888/pcd11.130338

**Published:** 2014-05-01

**Authors:** Sherri G. Homan, David G. McBride, Shumei Yun

**Affiliations:** Author Affiliations: Sherri G. Homan, David G. McBride, Missouri Department of Health and Senior Services, Jefferson City, Missouri.

## Abstract

**Introduction:**

The Well-Integrated Screening and Evaluation for Women Across the Nation (WISEWOMAN) public health program is designed to reduce the risk of heart disease and stroke among low-income, underinsured or uninsured women through clinical screenings, risk factor assessment, and lifestyle interventions. We assessed the effect of the Missouri WISEWOMAN program on the control of high blood pressure, total cholesterol, and blood glucose levels.

**Methods:**

We calculated the proportion of participants (N = 1,130) with abnormal blood pressure, total cholesterol, or blood glucose levels at an initial screening visit who gained control at a follow-up visit 11 to 18 months later during a 7-year period from June 30, 2005, to June 29, 2012. We used logistic regression to identify sociodemographic characteristics and other factors associated with achieving control.

**Results:**

Many WISEWOMAN participants gained control of their blood pressure (41.2%), total cholesterol (24.7%), or blood glucose levels (50.0%). After controlling for sociodemographic factors, smoking status, weight status, medication use, and number of lifestyle interventions, nondiabetic women with stage II hypertension (adjusted odds ratio [AOR] = 0.36, 95% confidence interval [CI] = 0.21–0.60) and diabetic women with stage I (AOR = 0.54, 95% CI = 0.32–0.92) and stage II (AOR = 0.23, 95% CI = 0.07–0.77) hypertension were less likely to achieve control of their blood pressure than nondiabetic women with stage I hypertension. Women aged 45 to 64, women with less than a high school education, women who were obese in the initial visit, women who gained 7% or more of their weight, and women who did not participate in any lifestyle intervention sessions were significantly less likely to achieve total cholesterol control than their counterparts.

**Conclusion:**

The Missouri WISEWOMAN program helps many participants achieve control of blood pressure, total cholesterol, and blood glucose levels; the lifestyle intervention is likely to help participants control total cholesterol. More efforts are needed for women with diabetes and stage II hypertension to achieve blood pressure control.

## Introduction

The Well-Integrated Screening and Evaluation for Women Across the Nation (WISEWOMAN) public health program is designed to reduce the risk of heart disease and stroke among low-income, underinsured, or uninsured women through clinical screenings, risk factor assessment, and lifestyle interventions (1,2). Missouri is among 20 states and tribal organizations that implement the WISEWOMAN program with the support of the Division for Heart Disease and Stroke Prevention at the Centers for Disease Control and Prevention (CDC) (3). Women with hypertension, high cholesterol, and high blood glucose are at serious health risk unless these conditions are controlled. The WISEWOMAN program was authorized by Congress in 1993 and began as a demonstration program in 1995. Evaluations so far have shown significant improvements in physical activity (4,5), dietary intake (6,7), and 10-year coronary heart disease risk (8,9) among WISEWOMAN participants receiving enhanced interventions versus usual-care or minimum interventions. WISEWOMAN participants at 1-year follow-up on average have improved blood pressure (1.3% systolic and 1.7% diastolic) and total blood cholesterol (2.0%) (10). WISEWOMAN participants with newly diagnosed diabetes have also shown significant improvements in blood glucose (11.5%), blood pressure (3.1%–3.5%), and total cholesterol (6.4%) at 1-year follow-up (11).

The purpose of this study was to assess the effect of the Missouri WISEWOMAN program on the control of high blood pressure, high total cholesterol, and elevated blood glucose at a follow-up visit 11 to 18 months after initial clinical examination. This is the first evaluation study of the Missouri WISEWOMAN program since its inception in 2003. This is one of few studies that focuses on effectiveness in terms of reaching normal limits of the biologic measures, which are more stringent than the percentage improvements (2,4,10). The Missouri Department of Health and Senior Services Institutional Review Board reviewed this project and found it to be exempt.

## Methods

### Program description

Women are eligible for the WISEWOMAN program if they are enrolled in Show Me Healthy Women (SMHW), the free breast and cervical cancer screening program in Missouri sponsored by the National Breast and Cervical Cancer Early Detection Program. Eligibility for SMHW includes having an annual household income at or below 200% of the federal poverty level and being aged 35 to 64 or older if a woman does not have Medicare Part B and no insurance to cover program services. Approximately 189 health care and local public health facilities recruit, enroll, or provide the SMHW screening services throughout the state, and approximately one-third of the SMHW providers also offer WISEWOMAN program services. The providers determine eligibility for each woman, and women meeting the eligibility criteria are eligible for all program services. Missouri WISEWOMAN program services include annual screenings of blood pressure, total cholesterol, high-density lipoprotein cholesterol, blood glucose, and height and weight for body mass index (BMI, calculated as weight in kilograms divided by height in meters squared [kg/m^2^]) determination. WISEWOMAN clients who are screened and have elevated levels reaching alert values for blood pressure (>180 mm Hg systolic or >110 mm Hg diastolic), blood total cholesterol (>400 mg/dL), or blood glucose (≤50 mg/dL or ≥275 mg/dL) receive a program-mandated referral for follow-up health services within 7 days and are monitored by the program. Referrals for women with elevated but nonalert values are encouraged but not required.

Clients are referred to attend a minimum of 3 lifestyle intervention (LSI) sessions with no limit on the maximum number of sessions. The LSI sessions are counseling and education sessions, each lasting 15 to 60 minutes, that focus on increasing physical activity, improving diet, and quitting smoking. Content of the sessions is based on the manual *A New Leaf . . . Choices for Healthy Living* (12); sessions are adjusted to clients’ individual needs and are conducted by using motivational interviewing techniques.

### Study population

This study included all WISEWOMAN program participants who had high blood pressure, total cholesterol, or blood glucose at the initial visit and who had a follow-up visit 11 to 18 months later from June 30, 2005 through June 29, 2012. A participant flow diagram illustrates the sample size at different enrollment points (Figure).

**Figure:**
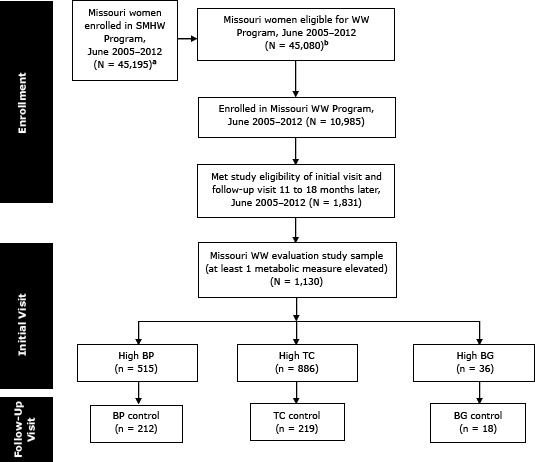
Flow diagram of the enrollment and evaluation process of the Missouri WISEWOMAN Program, June 2005 through June 2012. Abbreviations: SMHW, Show Me Healthy Women; WW, WISEWOMAN; BP, blood pressure; TC, total cholesterol; BG, blood glucose. ^a^ The number of women that received SMHW screening and/or diagnostic services. ^b^ The number of women that received SMHW screening only services and eligible for WISEWOMAN (WW) program services.

### Program data and study variables

Health history and clinical data from WISEWOMAN screenings and LSIs are collected and entered via the Internet into a real-time database system by health care provider staff. The system meets standards set for the Public Health Information Network and National Electronic Disease Surveillance System.

Race was self-reported by the clients, as 1 of 11 possible categories. Because of small numbers in some categories, race was summarized into 3 categories: African American, white, and other. Ethnicity (Hispanic or Non-Hispanic) was also self-reported, as was the highest education level attained. Age was calculated as the time elapsed from the client’s birth date to the initial visit date and placed into 10-year age groups of 35 to 44, 45 to 54, and 55 to 64 years. This categorization will help to evaluate whether the program has different effects on women of various age groups: young middle-aged, middle-aged, and older middle-aged women.

Smoking status was self-reported at each visit and grouped into 4 categories: smoking at each visit; nonsmoking at each visit; quitter, which indicated smoking at the initial visit but had quit by the follow-up visit; and new smoker, which indicated nonsmoking at the initial visit but initiated smoking before the follow-up visit. Weight status was defined by BMI: normal or low weight (BMI <25.0), overweight (BMI 25.0–29.9) or obese (BMI ≥30.0). Weight status change was the difference in weight from the initial visit to the follow-up visit and grouped into 5 categories: 1) weight loss of 7% or more, 2) weight loss of 2% or more but less than 7%, 3) weight loss of less than 2% to weight gain of less than 2%, 4) weight gain of 2% or more but less than 7%, or 5) weight gain of 7% or more. Medication status was self-reported current or periodic use of a medication to control the condition. The number of LSI sessions attended was grouped into 4 categories (0, 1 or 2, 3 or 4, or 5 or more).

During each visit, 2 blood pressure readings were taken, and the average was recorded. Stage I hypertension was defined as an average systolic blood pressure of 140 to 159 mm Hg or diastolic blood pressure of 90 to 99 mm Hg. For women with diabetes, it was defined as an average systolic blood pressure of 130 to 159 mm Hg or diastolic blood pressure of 80 to 99 mm Hg (13). Stage II hypertension was defined as an average systolic blood pressure of 160 mm Hg or more or a diastolic blood pressure of 100 mm Hg or more for all clients. Elevated blood pressure levels for diabetic clients were considered controlled when the client’s average blood pressure was less than 130 mm Hg for systolic and less than 80 mm Hg for diastolic. For nondiabetics, elevated blood pressure levels were considered controlled when the systolic pressure was less than 140 mm Hg and the diastolic pressure was less than 90 mm Hg. Elevated blood glucose was defined as fasting plasma glucose (FPG) level of 126 mg/dL or more; control was defined as FPG of less than 126 mg/dL (14) when pretest fasting for at least 9 hours. Clients with nonfasting glucose levels were excluded. Nonfasting total cholesterol concentration was used for this analysis. Borderline high total cholesterol was defined as 200 to 239 mg/dL, and high total cholesterol was 240 mg/dL or higher (15). A total cholesterol level of less than 200 mg/dL was considered to be controlled.

### Statistical analysis

We calculated the proportion of participants with high blood pressure, total cholesterol, or blood glucose at their initial visit who gained control at a follow-up visit 11 to 18 months later. The proportion was further stratified by age, race/ethnicity, education level, smoking status, weight status, weight change, initial blood pressure and total cholesterol, use of medication at follow-up visit, and number of LSI sessions participated in. Univariate logistic regression was used to test the association between these factors and blood pressure and total cholesterol control. Multivariate logistic regression was used to control for confounders. Statistical significance was determined by 95% confidence intervals (CIs) for the crude or adjusted odds ratios (AORs). There were too few cases to permit stratification and multivariate analysis for blood glucose.

## Results

### Participants

A total of 1,130 clients had at least 1 metabolic measure elevated at the initial visit and had a follow-up visit 11 to 18 months later (Table 1). Most participants self-reported as white (88.2%) and only 4.0% self-identified as Hispanic. Most clients (54.2%) reported having completed high school; most of the remaining clients were divided between less (23.6%) and more education (21.3%) (Table 1). Those proportions were consistent across age and racial categories. However, among Hispanic clients, 48.9% indicated less than a high school education, 35.6% indicated a high school education, and 15.6% indicated education beyond high school.

**Table 1:** Characteristics of Missouri WISEWOMAN Program Participants Included in This Study, 2005–2012

Characteristic	No. at Initial Visit (%)^a^	No. at Follow-Up Visit (%)^a^
**Overall^b^**	**1,130 (100.0)**	** —**
**Race**		
White	997 (88.2)	—
African American	94 (8.3)	—
Other	37 (3.3)	—
Unknown	2 (0.2)	—
**Ethnicity**		
Hispanic	45 (4.0)	—
Non-Hispanic	1,074 (95.0)	—
Unknown	11 (1.0)	—
**Age group, y**		
35–44	272 (24.1)	—
45–54	463 (41.0)	—
55–64	395 (35.0)	—
**Education**		
<High school diploma	267 (23.6)	—
High school diploma	613 (54.2)	—
>High school diploma	241 (21.3)	—
Unknown	9 (0.8)	—
**Smoking status**		
Current smoker	412 (36.5)	404 (35.8)
Nonsmoker	706 (62.5)	719 (63.6)
Unknown/refused	12 (1.1)	7 (0.6)
**Weight status (body mass index, kg/m^2^)**		
Normal weight (<25.0)	230 (20.4)	224 (19.8)
Overweight (25.0–29.9)	319 (28.2)	324 (28.7)
Obese (≥30.0)	579 (51.2)	573 (50.7)
Unknown (missing or out-of-range)	2 (0.2)	9 (0.8)
**Chronic disease/condition**		
High BP only	217 (19.2)	153 (13.5)
High TC only	590 (52.2)	463 (41.0)
High BG only	15 (1.3)	11 (1.0)
High BP and TC	262 (23.2)	232 (20.5)
High BP and BG	12 (1.1)	12 (1.1)
High TC and BG	10 (0.9)	14 (1.2)
High BP, TG, and BG	24 (2.1)	20 (1.8)
**Use of medication for condition**s		
Taking BP-lowering medication among those with abnormal BP	204 (39.6)	252 (48.9)
Taking cholesterol-lowering medication among those with high TC	73 (8.2)	133 (15.0)
Taking BG-lowering medication among those with high BG	22 (36.1)	37 (60.7)
**No. of lifestyle intervention sessions^c^**		
0	—	45 (4.0)
1 or 2	—	843 (74.6)
3 or 4	—	217 (19.2)
≥5	—	25 (2.2)

Abbreviations: — , same value as initial visit or not applicable; BP, blood pressure; TC, total cholesterol; BG, blood glucose.

a Percentages may not sum to 100% because of rounding.

b Participants with at least 1 uncontrolled measure.

c Number of lifestyle intervention sessions attended from initial to follow-up visit.

Approximately 27.3% of the women had multiple chronic conditions at the initial visit, compared with 24.6% at the follow-up visit. Approximately 39.6% of those with high blood pressure took blood pressure–lowering medication at the initial visit, compared with 48.9% at the follow-up visit. The percentage of participants with high total cholesterol who were taking cholesterol-lowering medication also increased from 8.2% at the initial visit to 15.0% at the follow-up visit. Among women with elevated blood glucose the percentage taking glucose-lowering medication increased from 36.1% to 60.7% (Table 1).

### High blood pressure control

A substantial proportion of Missouri WISEWOMAN program participants with uncontrolled high blood pressure at the initial visit gained control at the follow-up visit (41.2%) (Table 2). The proportion gaining control was significantly lower among nondiabetic women with stage II hypertension (27.7%, OR = 0.39, 95% CI = 0.25–0.62) and diabetic women with stage I (36.6%, OR = 0.58, 95% CI = 0.36–0.95) and stage II (21.1%, OR = 0.27, 95% CI = 0.09–0.84) hypertension than among nondiabetic women with stage I hypertension (49.6%). After adjusting for sociodemographic characteristics and all other factors listed in Table 2, nondiabetic women with stage II hypertension (AOR = 0.36, 95% CI = 0.21–0.60) and diabetic women with stage I (AOR = 0.54, 95% CI = 0.32–0.92) and stage II (AOR = 0.23, 95% CI = 0.07–0.77) hypertension were significantly less likely to gain control of their blood pressure than nondiabetic women with stage I hypertension. No other factors, including the number of LSI sessions and use of medication, were associated with blood pressure control.

**Table 2:** Blood Pressure (BP) Control at Follow-Up Visit Among Missouri WISEWOMAN Program Participants With High BP at Initial Visit, 2005–2012

Characteristic	Number^a^	% With Controlled BP	Odds Ratio (95% CI)	Adjusted OR^b^ (95% CI)
**Overall^c^**	515	41.2	—	—
**Race**				
White	444	42.6	1 [Reference]	
African-American	54	40.5	0.93 (0.52–1.65)	1.08 (0.57–2.04)
Other	15	60.0	2.20 (0.77–6.29)	2.42 (0.72–8.11)
**Ethnicity**				
Non-Hispanic	498	41.6	1.23 (0.91–1.66)	3.88 (0.09–16.9)
Hispanic	17	29.4	1 [Reference]	
**Age group, y**				
35–44	98	46.9	1 [Reference]	
45–54	208	40.9	0.78 (0.48–1.27)	0.80 (0.47–1.38)
55–64	209	38.8	0.72 (0.44–1.16)	0.88 (0.50–1.53)
**Education**				
<High school diploma	126	37.3	1 [Reference]	
High school diploma	280	40.7	1.15 (0.75–1.78)	1.18 (0.73–1.90)
>High school diploma	103	49.5	1.65 (0.97–2.80)	1.66 (0.92–3.00)
**Smoking status**				
Smoking at each visit	153	41.8	1 [Reference]	
Nonsmoking at each visit	332	38.6	0.87 (0.59–1.29)	0.95 (0.61–1.48)
Quitter	14	64.3	2.50 (0.80–7.82)	2.39 (0.70–8.18)
New smoker	9	77.8	4.87 (0.98–24.2)	5.35 (0.97–29.59)
**Weight status (body mass index, kg/m^2^)**				
Normal weight (<25.0)	77	44.2	1 [Reference]	
Overweight (25.0–29.9)	120	39.2	0.81 (0.46–1.46)	0.82 (0.43–1.54)
Obese (≥30.0)	317	41.3	0.89 (0.54–1.47)	0.96 (0.54–1.71)
**Weight change**				
Weight loss ≥7%	64	47.8	1.44 (0.70–2.98)	1.88 (0.81–4.35)
Weight loss of 2% or more but less than 7%	114	43.9	1.45 (0.76–2.78)	1.96 (0.94–4.10)
Weight loss of less than 2% to weight gain of less than 2%	178	38.8	1.18 (0.64–2.18)	1.55 (0.76–3.12)
Weight gain of 2% or more but less than 7%	99	44.4	1.49 (0.77–2.88)	1.81 (0.85–3.84)
Weight gain ≥7%	60	35.0	1 [Reference]	
**Initial blood pressure range, systolic/diastolic, mm Hg^d^**				
140/90–159/99 (nondiabetic, stage I)	284	49.6	1 [Reference]	
130/80–159/99 (diabetic, stage I)	93	36.6	0.58 (0.36–0.95)	0.54 (0.32–0.92)
≥160/100 (nondiabetic, stage II)	119	27.7	0.39 (0.25–0.62)	0.36 (0.21–0.60)
≥160/100 (diabetic, stage II)	19	21.1	0.27 (0.09–0.84)	0.23 (0.07–0.77)
**Use of medication at follow-up visit**				
Yes	252	40.1	0.85 (0.59–1.23)	1.16 (0.76–1.78)
Yes but not on the day of follow-up visit	32	34.4	0.67 (0.31–1.45)	0.74 (0.32–1.73)
No	223	44.0	1 [Reference]	
**No. of lifestyle intervention sessions**				
0	25	32.0	1 [Reference]	
1 or 2	363	41.9	1.53 (0.64–3.64)	1.93 (0.74–5.05)
3 or 4	107	42.1	1.54 (0.61–3.89)	2.33 (0.83–6.55)
≥5	20	35.0	1.14 (0.33–3.97)	1.62 (0.46–7.14)

Abbreviations: OR, odds ratio; CI, confidence interval; — , not applicable.

a Number of participants who have high blood pressure at initial visit and have blood pressure data at follow-up visit.

b Adjusted for all other factors in the table. For the effect of the number of lifestyle intervention sessions attended, weight status change was not controlled because it could be an intermediate variable; including weight change in the model had little effect on ORs and 95% CIs.

c Initial blood pressure average ≥140/90 (no diabetes) or 130/80 (diabetes).

d Average initial blood pressure and stage of hypertension.

### High total cholesterol control

Overall, approximately one-fourth (24.7%) of women with high total cholesterol at the initial visit achieved control by the follow-up visit (Table 3). A significantly lower percentage of middle aged and older women (23.3% among those age 45–54 years, OR = 0.62, 95% CI = 0.43–0.91; 20.8% among those aged 55–64 years, OR = 0.54, 95% CI = 0.36–0.81) achieved cholesterol control compared with women aged 35 to 44 (32.7%). A significantly lower percentage of women whose total cholesterol on initial exam was high, at 240 mg/dL or greater (11.9%, OR = 0.30, 95% CI = 0.20–0.44), achieved cholesterol control compared with women with borderline high total cholesterol levels of 200 to 239 mg/dL (31.4%). Women who reported use of cholesterol-lowering medication at the follow-up visit were significantly more likely to gain control (41.4% for those using medication at follow-up visit, OR = 2.66, 95% CI =1.80–3.93; 45.5% for those using medication but not on the day of the follow-up visit, OR = 3.14, 95% CI = 1.33–7.42) than those who did not use medication at the time of the visit (21.0%).

**Table 3:** Blood Cholesterol Control at Follow-Up Visit, Among Missouri WISEWOMAN Program Participants With Elevated Total Cholesterol (TC) at Initial Visit, 2005–2012

Characteristic	Number^a^	% With Controlled TC	OR (95% CI)	Adjusted OR^b^ (95% CI)
**Overall** c	886	24.7	—	—
**Race**				
White	791	24.5	1 [Reference]	
African American	60	23.3	0.94 (0.50–1.74)	0.71 (0.38–1.31)
Other	33	27.3	1.15 (0.53–2.53)	1.54 (0.65–3.62)
**Ethnicity**				
Non-Hispanic	849	24.6	1 [Reference]	
Hispanic	37	27.0	1.13 (0.54–2.38)	1.20 (0.57–2.54)
**Age group, y**				
35–44	214	32.7	1 [Reference]	
45–54	365	23.3	0.62 (0.43–0.91)	0.49 (0.31–0.76)
55–64	307	20.8	0.54 (0.36–0.81)	0.43 (0.27–0.69)
**Education**				
<High school diploma	214	25.7	1 [Reference]	
High school diploma	475	22.9	0.86 (0.59–1.25)	1.4 (0.97–2.03)
>High school diploma	191	28.3	1.14 (0.73–1.77)	2.20 (1.37–3.53)
**Smoking status change**				
Smoking in both visit	306	28.4	1 [Reference]	
Nonsmoking at both visits	527	21.6	0.70 (0.50–0.96)	0.88 (0.62–1.24)
Quitter	21	42.9	1.87 (0.77–4.64)	1.86 (0.56–6.14)
New smoker	16	25.0	0.84 (0.26–2.67)	5.30 (0.66–42.46)
**Weight status (body mass index, kg/m^2^)**				
Normal weight (<25.0)	193	21.2	1 [Reference]	
Overweight (25.0–29.9)	262	24.0	1.17 (0.75–1.83)	0.65 (0.41–1.04)
Obese (≥30.0)	430	26.7	1.35 (0.90–2.03)	0.40 (0.26–0.61)
**Weight change**				
Weight loss ≥7%	103	28.2	1.44 (0.76–2.73)	1.96 (1.02–3.77)
2% ≤ weight loss <7%	209	25.4	1.25 (0.71–2.20)	1.89 (1.09–3.30)
−2% < weight change <2%	275	24.7	1.21 (0.70–2.09)	1.25 (0.74–2.09)
2% ≤ weight gain <7%	196	24.0	1.16 (0.65–2.06)	1.57 (0.91–2.73)
Weight gain ≥7%	103	21.4	1 [Reference]	
**Total cholesterol, mg/dL**				
200–239 (borderline high)	583	31.4	1 [Reference]	
≥240 (high)	303	11.9	0.30 (0.20–0.44)	1.04 (0.75–1.45)
**Use of medication at follow-up visit**				
Yes	133	41.4	2.66 (1.80–3.93)	0.69 (0.46–1.05)
Yes but not on the day of follow-up visit	22	45.5	3.14 (1.33–7.42)	0.84 (0.33–2.14)
No medication	711	21.0	1 [Reference]	
**No. of lifestyle intervention sessions**				
0	37	21.6	1 [Reference]	
1 or 2	669	24.1	1.15 (0.52–2.56)	3.07 (1.48–6.35)
3 or 4	167	26.9	1.34 (0.57–3.14)	3.16 (1.43–6.99)
≥5	13	38.5	2.27 (0.58–8.87)	3.27 (0.77–13.87)

Abbreviations: OR, odds ratio; CI, confidence interval; — , not applicable.

a Number of participants who had high total blood cholesterol at initial visit and had total blood cholesterol data at follow-up visit.

b Adjusted for all other factors in the table. For the effect of the number of lifestyle intervention sessions attended, weight status change was not controlled because it could be an intermediate variable; including weight change in the model had little effect on ORs and 95% CIs.

c Initial visit total cholesterol ≥200 mg/dL.

After controlling for sociodemographic characteristics and other variables included in Table 3, middle-aged and older women (AOR = 0.49, 95% CI = 0.31–0.76 for those aged 45–54 years; AOR = 0.43, 95% CI = 0.27–0.69 for those aged 55–64 years) were less likely to achieve total cholesterol control than women aged 35 to 44 years. Women with more than a high school education were more likely to gain control than those with less than a high school education (AOR = 2.20, 95% CI = 1.37–3.53). Women who were obese at the initial visit were less likely to achieve control (AOR = 0.40, 95% CI = 0.26–0.61) than women at a normal weight at the initial visit. Women who lost 2% or more body weight between the initial and the follow-up visit were more likely to gain control (AOR = 1.89, 95% CI = 1.09–3.30 for those who lost 2% or more but less than 7%; AOR = 1.96, 95% CI = 1.02–3.77 for those who lost 7% or more body weight) than those who gained 7% or more weight. Women who participated in 1 or more LSI sessions were more likely to gain control (AOR = 3.07, 95% CI = 1.48–6.35 for those who participated in 1 or 2 sessions; AOR = 3.16, 95% CI = 1.43–6.99 for those participated in 3 or 4 sessions) than those who did not participate in any session.

### Elevated blood glucose control

Of the 36 women with elevated blood glucose who met the criteria for this study, 18 (50.0%) gained control of their blood glucose by the follow-up visit. All clients in this analysis with elevated blood glucose attended at least one LSI session.

## Discussion

Although 1-year follow-up evaluations of the WISEWOMAN program have been published, most have compared change in physical activity, diet, and percentage improvement in blood pressure, blood cholesterol, and cardiovascular disease (CVD) risk (2,4,10). This is one of few studies that focuses on effectiveness in terms of reaching normal limits of blood pressure, total cholesterol, and blood glucose. Therefore, the study outcome in our evaluation is more stringent than outcomes in other studies that investigate percentage improvement (2,4,10). Although percentage improvement is a sensitive measure of program effect (eg, 50% of participants who had abnormal total cholesterol at the initial visit improved their total cholesterol by 5%), the percentage of participants that gain control is a direct measure if the goal of the program has been reached.

Our study found that many participants in the Missouri WISEWOMAN program gained control of blood pressure, total cholesterol, and blood glucose at 1-year follow-up. Gaining control of these risk factors may postpone adverse effects and lead to decreased morbidity and mortality rates from coronary heart disease (16). Meanwhile, we also found that a high proportion of participants (ie, 58.8% with high blood pressure, 75.3% with high total cholesterol, and 50.0% with elevated blood glucose) did not gain control at 1-year follow-up. Therefore, the program should identity effective strategies to help a broader range of participants to control their blood pressure, cholesterol, and glucose.

To assist with identifying intervention strategies, we studied the factors associated with gaining control or lack of control. We found that women with diabetes or a higher stage of hypertension were less likely to gain control of their blood pressure. Use of medication at the follow-up visit was not associated with greater control of hypertension than no medication use. This finding suggests that adherence to medication regimens is low, the medication regimens participants are taking are not optimal, or the self-reported data on medication use are inaccurate. As mentioned, the program is required to provide referral for participants who have alert levels of blood pressure, cholesterol, or glucose. However, because the program does not cover treatment, its effect on medication use is likely to be limited. Meanwhile, many social, economic, and behavioral attributes of participants that may affect control were not measured and therefore unavailable for analysis as possible predictor variables.

For blood cholesterol, we found that younger women were more likely to gain control than older women, after controlling for other factors. Similarly, women who were at a normal weight at the initial visit versus those who were obese, those who lost 2% or more weight versus those who gained 7% or more weight, and those who had participated in 1 or more LSI sessions versus those who had not participated in any were more likely to gain control. Use of medication was associated with increased control in crude analysis; however, after controlling for other factors, the association was not significant. This may have been due in part to small study size and possibly as a result of some correlation between medication use and body weight change or between medication use and lifestyle intervention factors.

There were some limitations in this study. First, there was no comparison group. Therefore, determining whether the improvement was completely a result of the program is not possible. However, we believe the program was at least partially responsible for the findings. Second, lifestyle factors and medication use information were self-reported and may not be accurate. Third, the study size was small and included 10.2% of all WISEWOMAN participants; however, even if we extended the period for the follow-up visit to up to 3 years after the initial visit, it would only cover 20.9% of all WISEWOMAN participants. The reasons women do not return for a follow-up visit are unknown and can be further studied. However, the women in our study tended to be older, white, and more educated than the women not returning for a follow-up visit. Finally, the study used stringent measures of health outcomes, which are less sensitive than percentage improvement of blood pressure, total cholesterol, and blood glucose used in other studies but more relevant to the program’s main goal of preventing heart disease. Therefore, this limitation could also be considered a strength of the study.

Regardless of these limitations, we used initial and follow-up data to study the effect of the Missouri WISEWOMAN program on the control of blood pressure, total cholesterol, and blood glucose levels and the association between control and multiple sociodemographic and other factors. This is the first study conducted since the inception of the Missouri WISEWOMAN program, and it will help guide future program planning and implementation.

CVD is the number one killer in Missouri, as well as in the United States (17). High blood pressure, high blood cholesterol, and elevated blood glucose are risk factors for CVD and are widely recognized as critical public health issues among women in Missouri, especially among women of lower socioeconomic status. The WISEWOMAN program provides screening services for all participants and referral services for those with alert levels of blood pressure, cholesterol, and glucose. Findings from this study indicate that the Missouri WISEWOMAN program is associated with improvements in control of blood pressure, cholesterol, and glucose. The LSI sessions are likely to help control total cholesterol. More efforts are needed to focus on profiles of women who did not achieve control: those with diabetes and stage II hypertension for blood pressure and women aged 45 to 64 years, women with less than a high school education, and obese women for total cholesterol.
